# The incidence of collagen-associated adverse events in pediatric population with the use of fluoroquinolones: a nationwide cohort study in Taiwan

**DOI:** 10.1186/s12887-020-1962-0

**Published:** 2020-02-11

**Authors:** Pei-Han Yu, Chih-Fen Hu, Jen-Wei Liu, Chi-Hsiang Chung, Yong-Chen Chen, Chien-An Sun, Wu-Chien Chien

**Affiliations:** 1 Master Program of Big Data in Biomedicine, College of Medicine, Fu Jen Catholic University, New Taipei City, Taiwan; 2 Department of Pharmacy, Fu Jen Catholic University Hospital, Fu Jen Catholic University, New Taipei City, Taiwan; 3 Department of Pediatrics, Tri-Service General Hospital, National Defense Medical Center, Taipei, Taiwan; 4 School of Pharmacy, College of Pharmacy, Taipei Medical University, Taipei, Taiwan; 5 School of Public Health, National Defense Medical Center, Taipei, 11490 Taiwan; 6 School of Medicine, College of Medicine, Fu Jen Catholic University, New Taipei, Taiwan; 7 Big Data Research Center, College of Medicine, Fu Jen Catholic University, New Taipei City, Taiwan; 8 Department of Public Health, College of Medicine, Fu Jen Catholic University, New Taipei City, Taiwan; 9 Graduate Institute of Life Sciences, National Defense Medical Center, Taipei, 11490 Taiwan; 10 Department of Medical Research, Tri-Service General Hospital, National Defense Medical Center, No 325, Section 2, Cheng-Kung Road, Taipei, 11490 Taiwan, Republic of China

**Keywords:** Fluoroquinolones, Pediatric patients, Collagen-associated adverse effects, Prescription safety issue

## Abstract

**Background:**

To evaluate the safety of using fluoroquinolones in pediatric population in Taiwan.

**Methods:**

Patients aged 0~18 years old with fluoroquinolones prescriptions ≥5 consecutive days during year 2000 to 2013 were selected from the National Health Insurance Research Database, 4-time case number were selected as controls. We evaluated the patient’s outcome after the use of fluoroquinolones by reviewing a newly diagnosis of the following collagen-associated adverse events by International Classification of Diseases, Ninth Revision, Clinical Modification codes, covering tendons rupture, retinal detachments, gastrointestinal tract perforation, aortic aneurysm or dissection.

**Results:**

Of the enrolled patients (*n* = 167,105), collagen-associated adverse effects developed in 85 cases (0.051%) in 6-month tracking, including 0.051% in the fluoroquinolones study cohort (17 in 33,421) and 0.051% (68 in 133,684) in the fluoroquinolones free comparison cohort. The crude hazard ratio for collagen-associated adverse events in the fluoroquinolones group was 0.997 (0.586–1.696; *p* = 0.990). After adjusting for age, sex, catastrophic illness, low-income household, seasons, levels of urbanization, and healthcare, the corrected hazard ratio in 6-month tracking with FQs was 1.330 (95% CI; 0.778–2.276; *p* = 0.255).

**Conclusions:**

There is no significant difference of collagen-associated adverse effects between fluoroquinolones group and fluoroquinolones free group from our data. We propose that fluoroquinolones for pediatric population in clinical practice may be not so harmful as previous references reported.

## Background

Fluoroquinolones (FQs) are effective antimicrobial agents by directly inhibiting the process of bacterial DNA synthesis. With the broad-spectrum antibacterial coverage, they are widely used in bacterial infections, like acute bacterial sinusitis, acute bacterial exacerbations of chronic bronchitis, and uncomplicated urinary tract infections for adults. In addition to the strength of broad-spectrum antibacterial coverage, other advantages, such as high oral bioavailability, large volume of distribution, ideal tissue penetration and long-lasting medicine, make FQs a favorable choice in treating infectious diseases [1, 2].

However, FQs are not the first choice in clinical guideline for treating infectious diseases, since they have been reported to be associated with collagen degradation, which may lead to severe and detrimental adverse effects like tendon ruptures, retinal detachments, gastrointestinal tract perforation, even aortic aneurysms in adults [3–7]. FQs were previously found to cause apoptotic changes in extracellular matrix and significantly decrease collagen type I and the β1-integrin receptors [8]. Collagen defects-related tissue damages are found not only in tendons, but also tissues composed of collagen, such as aortic wall, gastrointestinal (GI) tract, and retina [5, 9, 10]. All of these findings attribute to the cause of collagen-associated adverse effects. To alarm the risks of adverse effects of FQs, the U.S. FDA declared several warnings containing potential tendinitis, tendon ruptures, and even irreversible peripheral neuropathy [11, 12].

On the other hand, the original toxicological studies with quinolones documented the cartilage injury in weight-bearing joints in canine puppies [13]. With the concern of potential negative impacts on musculoskeletal development in growing children, systemically administered FQs are not recommended for routine use in children younger than18 years old except for some complicated and complex infectious diseases cases [14]. To follow the clinical guidelines and recommendations in treating infectious diseases in children [15], the physicians tend to reserve the prescriptions of FQs as the last line antibiotics to tackle with fulminant and invasive bacterial infections at hospitals in Taiwan.

Although the use of FQs to treat bacterial infection in pediatric population has been decreasing with time, there is still a small pediatric group who needs FQs for treating serious and multiple drug-resistant bacterial infections rising in the community [16, 17]. For this reason, it is urgently needed to evaluate the safety of FQs in clinical use. After reviewing the literatures, we found that most of the study subjects for adverse effects of FQs were older than 18-year old patients [3–5, 7, 18]. By contrast, there are still a few publications reporting benefits for using FQs in children [19, 20]. Since the lack of sufficient evidence-based articles found in pediatric population, the purpose of our study is to investigate the safety issue of FQs-induced collagen-associated adverse effects at the young age group.

## Methods

### Data sources

We performed a cohort study by using the National Health Insurance Research Database (NHIRD) of Taiwan. The National Health Insurance (NHI) system of Taiwan covers more than 99.6% of the Taiwanese population [21]. This database is representative of the population in Taiwan, and always used in generating evidence to support clinical decisions or healthcare policy-making [22]. All data from primary outpatient departments and inpatient hospital care settings after year 2000 were included in this database. This longitudinal health insurance database in year 2000–2013 in Taiwan was collected by randomly selecting individuals from the registry for beneficiaries of the NHI program. It contains complete outpatient and inpatient electronic claim records, individual diagnoses, procedures, and medicine prescription. This database is commonly used in several population-based studies and pharmacoepidemiologic research in Taiwan. (https://nhird.nhri.org.tw/).

### Medication exposure

In this study, medication exposure was defined as receiving FQs in either oral or intravenous form of the following active compounds including ciprofloxacin, levofloxacin, ofloxacin, gemifloxacin, norfloxacin, and moxifloxacin. Topical or external form of prescription was not included. The prescription period was equal to (or longer than) five consecutive days to correspond with the inclusion criteria.

### Inclusion and exclusion criteria

**Fig. 1:**
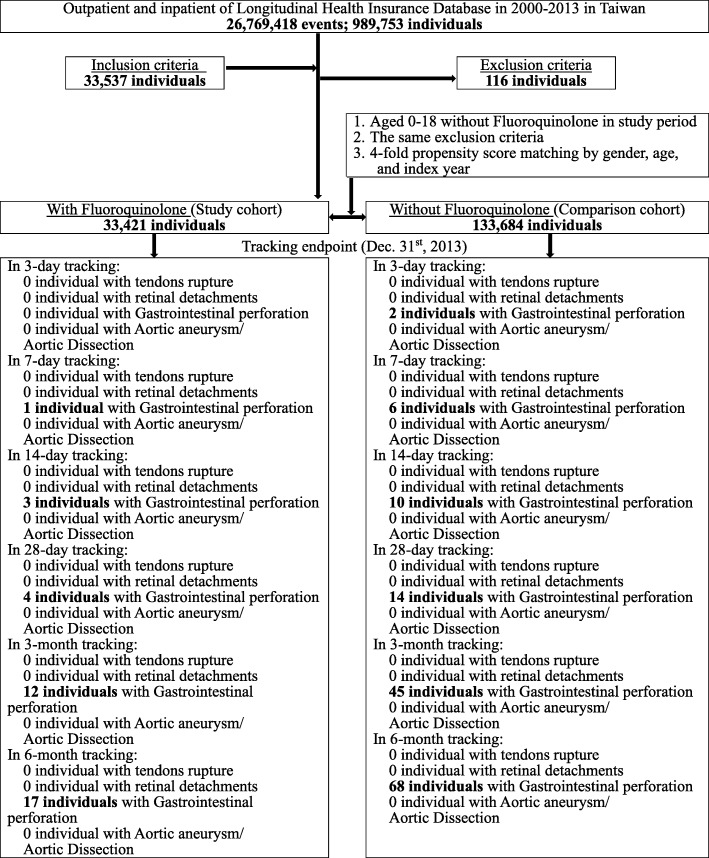
The flowchart of study sample selections

We utilized data between year 2000 and 2013 from NHIRD of a sub-dataset, longitudinal health insurance database (*n* = 989,753). All patients aged 0~18 years old with FQs prescriptions ≥5 consecutive days after January 1, 2000 were selected and included into this cohort. The total cases were 33,537 individuals included. The patients without tracking information (*n* = 22), with missing data on gender (*n* = 7), those who ever received FQs before index date (*n* = 65), with collagen-associated adverse events, and primary or secondary collagen diseases prior to enrollment (*n* = 4) were excluded. Also, we excluded cases with the diagnosis of appendicitis (International Classification of Diseases, Ninth Revision, Clinical Modification (ICD-9-CM code): 540–543), peritonitis (ICD-9-CM code: 567), and typhoid fever (ICD-9-CM code: 002) (*n* = 18) because the symptoms of these diseases are similar to one of the primary outcomes (GI perforation) in this study [4]. The total excluded cases were 116 individuals. Overall, the final enrolled cases with the use of FQs were 33,421 individuals. Propensity score matching was used for selecting control group, and 4-fold case number were selected (Fig. 1).

### Outcome

All of the diseases and adverse effects were defined by ICD-9-CM. The outcome was evaluated and defined by the newly diagnosis of the collagen-associated adverse events, including tendons rupture (727.6), retinal detachments (361.0), gastric perforation (531.1, 531.2, 531.5, 531.6, 532.1, 532.2, 532.5, 532.6, 533.1, 533.2, 533.5, 533.6, 534.1, 534.2, 534.5, and 534.6), small or large intestinal perforation (569.83) [4], aortic aneurysm (441.1, 441.2, 441.3, 441.4, 441.5, 441.6, 441.7, and 441.9), and aortic dissection (441.0, 441.00, 441.01, 441.02, and 441.03) [18].

### Covariates

In order to be as comprehensive as possible in adjusting for factors that might confound the studied association, we identified several covariates such as catastrophic illness to adjust the baseline health conditions of selected patients, and low-income household for individual financial conditions. On the other hand, medicine such as nonsteroidal anti-inflammatory drugs (NSAIDs) and steroid are both well-known for causing gastrointestinal mucosal damage [23, 24]. Since these two medicines were commonly used in our study population, we added these two items as covariates to eliminate the interference of outcome interpretation. Moreover, we also adjusted for selected other variables such as seasons, levels of urbanization, healthcare, and comorbidities (including cerebrovascular accident, diabetes Mellitus, hypertension, hyperlipidemia) [25–27] for minimizing potential biases.

### Statistical analysis

Statistical analyses were performed using Chi-square/Fisher exact test on category variables and t-test on continuous variables. Characteristics and outcome events of cohort patients for the FQs and FQs free groups were reported as number and percentage, mean and standard deviation, as appropriate. The Kaplan–Meier analysis and log-rank test were used for calculating the cumulative incidence rates of collagen-associated adverse events between FQs group and FQs-free group. The multivariable Cox proportional hazard model was used to estimate the hazard ratio (HR) of collagen-associated adverse events associated with the use of FQs. Our definition of significant level was 0.05 to detect differences in collagen-associated adverse events between FQs group and FQs-free group. All analyses were performed with SPSS version 22.

## Results

This study involved a total of 33,421 for the FQs group and 133,684 for the FQs free controls. Of the enrolled patients (*n* = 167,105), collagen-associate adverse effects developed in 85 (0.051%) in 6-month tracking, including 0.051% in the FQs study cohort (17 in 33,421) and 0.051% (68 in 133,684) in the FQs-free comparison cohort.

### Baseline characteristics of patients

Baseline characteristics of case patients and comparators are listed in the Table 1. There were no significant differences between the FQs group and FQ-free group in the distributions of age and sex, but the proportion of catastrophic illness was slightly higher in the FQs group than the FQs-free groups (0.6 vs. 0.38).

**Table 1:** Characteristics of study

Fluoroquinolones	Total		With		Without		*P*
Variables	n	%	n	%	n	%	
Total	167,105		33,421	20.00	133,684	80.00	
Collagen-associated adverse events							0.990
Without	167,020	99.95	33,404	99.95	133,616	99.95	
With	85	0.05	17	0.05	68	0.05	
Collagen-associated adverse events subgroups							0.990
Without	167,020	99.95	33,404	99.95	133,616	99.95	
Tendons rupture	0	0.00	0	0.00	0	0.00	
Retinal detachments	0	0.00	0	0.00	0	0.00	
Gastrointestinal perforation	85	0.05	17	0.05	68	0.05	
Aortic dissection/Aortic aneurysm	0	0.00	0	0.00	0	0.00	
Gender							0.999
Male	85,990	51.46	17,198	51.46	68,792	51.46	
Female	81,115	48.54	16,223	48.54	64,892	48.54	
Age (years)	9.78 ± 5.58		9.83 ± 5.35		9.77 ± 5.63		0.079
Age group (years)							0.999
< 1	5100	3.05	1020	3.05	4080	3.05	
1	7145	4.28	1429	4.28	5716	4.28	
2	7970	4.77	1594	4.77	6376	4.77	
3	9060	5.42	1812	5.42	7248	5.42	
4	10,680	6.39	2136	6.39	8544	6.39	
5–9	46,670	27.93	9334	27.93	37,336	27.93	
10–14	42,785	25.60	8557	25.60	34,228	25.60	
≧15	37,695	22.56	7539	22.56	30,156	22.56	
Catastrophic illness							< 0.001
Without	166,398	99.58	33,222	99.40	133,176	99.62	
With	707	0.42	199	0.60	508	0.38	
Low-income household							< 0.001
Without	164,089	98.20	32,561	97.43	131,528	98.39	
With	3016	1.80	860	2.57	2156	1.61	
Season							< 0.001
Spring (Mar - May)	43,867	26.25	8757	26.20	35,110	26.26	
Summer (Jun - Aug)	42,836	25.63	8435	25.24	34,401	25.73	
Autumn (Sep - Nov)	41,287	24.71	8289	24.80	32,998	24.68	
Winter (Dec - Feb)	39,115	23.41	7940	23.76	31,175	23.32	
Urbanization level							< 0.001
1 (the highest)	45,240	27.07	7343	21.97	37,897	28.35	
2	62,222	37.24	11,929	35.69	50,293	37.62	
3	27,980	16.74	6433	19.25	21,547	16.12	
4 (the lowest)	31,663	18.95	7716	23.09	23,947	17.91	
Level of care							< 0.001
Hospital center	7559	4.52	1268	3.79	6291	4.71	
Regional hospital	11,858	7.10	1939	5.80	9919	7.42	
Local hospital	8762	5.49	1588	4.94	7174	5.63	
Physician clinics	138,926	83.14	28,626	85.65	110,300	82.51	
Comorbidities							
Cerebrovascular accident	43	0.03	8	0.02	35	0.03	0.819
Diabetes mellitus	84	0.05	18	0.05	66	0.05	0.743
Hypertension	96	0.06	20	0.06	76	0.06	0.838
Hyperlipidemia	74	0.04	15	0.04	59	0.04	0.954
Drugs							
Non-steroidal anti-inflammatory drugs	5008	3.00	1023	3.06	3985	2.98	0.443
Systemic steroid	915	0.55	188	0.56	727	0.54	0.679

*P:* Chi-square / Fisher exact test on category variables and t-test on continue variables

### Collagen-associated adverse events

In the cohort, we did not observe any case of tendons rupture, retinal detachments, aortic aneurysms, and aortic dissection. Patients with collagen-associated adverse events (GI perforation) in FQs group included 1 individual in 7-day tracking, 3 individuals in 14-days tracking, 4 individuals in 28-days tracking, 12 individuals in 3-month tracking, and 17 individuals in 6-month tracking. However, patients with collagen-associated adverse events (GI perforation) in FQs free group were 2 individuals in 3-days tracking, 6 individuals in 7-days tracking, 10 individuals in 14-days tracking, 14 individuals in 28-days tracking, 45 individuals in 3-month tracking, 68 individuals in 6-month tracking (Fig. 1). The crude HR for collagen-associated adverse events in FQs group was 0.997 (95% CIs; 0.586–1.696; *p* = 0.990) (Table 2).

**Table 2:** Factors of gastrointestinal perforation in 6-month tracking by using Cox regression

Variables	Crude HR	95% CI	95% CI	*P*	Adjusted HR	95% CI	95% CI	*P*	Adjusted HR	95% CI	95% CI	*P*
Fluoroquinolones												
Without	Reference				Reference				Reference			
With	0.997	0.586	1.696	0.990	1.329	0.776	2.274	0.257	1.330	0.778	2.276	0.255
Gender												
Male	1.153	0.753	1.764	0.513	1.040	0.791	1.340	0.062	1.033	0.809	1.382	0.064
Female	Reference				Reference				Reference			
Age (years)	0.976	0.939	1.014	0.214	0.968	0.931	1.006	0.094				
Age group (years)												
< 1	Reference								Reference			
1	1.835	0.708	4.756	0.212					2.047	0.784	5.345	0.123
2	0.620	0.106	2.290	0.473					0.669	0.810	2.488	0.556
3	1.802	0.715	4.540	0.212					1.822	0.752	4.648	0.189
4	0.368	0.079	1.703	0.201					0.381	0.880	1.778	0.225
5–9	0.441	0.193	1.008	0.052					0.433	0.179	0.952	0.040
10–14	0.415	0.177	0.971	0.043					0.386	0.163	0.909	0.028
≧15	0.944	0.442	2.014	0.881					0.831	0.384	1.789	0.661
Catastrophic illness												
Without	Reference				Reference				Reference			
With	5.972	0.014	161.782	0.985	2.740	0.292	13.421	0.928	2.739	0.291	13.411	0.938
Low-income household												
Without	Reference				Reference				Reference			
With	1.643	0.090	4.620	0.661	1.546	0.076	4.105	0.578	1.541	0.072	4.070	0.572
Season												
Spring	Reference				Reference				Reference			
Summer	0.218	0.127	0.377	< 0.001	0.209	0.121	0.362	< 0.001	0.022	0.126	0.374	< 0.001
Autumn	0.017	0.006	0.047	< 0.001	0.018	0.006	0.048	< 0.001	0.017	0.007	0.050	< 0.001
Winter	0.056	0.032	0.098	< 0.001	0.057	0.032	0.099	< 0.001	0.057	0.032	0.100	< 0.001
Urbanization level												
1 (the highest)	1.993	1.032	3.849	0.040	2.034	1.033	4.002	0.033	2.043	1.031	4.024	0.029
2	0.596	0.276	1.288	0.188	0.526	0.239	1.144	0.115	0.527	0.242	1.146	0.116
3	2.357	1.184	4.691	0.015	2.581	1.294	5.146	0.003	2.586	1.294	5.153	0.006
4 (the lowest)	Reference				Reference				Reference			
Level of care												
Hospital center	2.909	1.232	6.871	0.015	2.768	1.132	6.759	0.014	2.757	1.124	6.734	0.010
Regional hospital	7.613	4.608	12.579	< 0.001	8.351	5.008	13.884	< 0.001	8.480	5.029	14.208	< 0.001
Local hospital	6.213	3.425	11.269	< 0.001	5.304	2.911	9.672	< 0.001	5.222	2.864	9.526	< 0.001
Physician clinics	Reference				Reference				Reference			
Comorbidities (No reference)												
Cerebrovascular accident	10.454	0.022	25.124	0.986	5.131	0.009	16.752	0.992	5.104	0.009	16.641	0.992
Diabetes mellitus	3.401	0.564	6.785	0.298	3.105	0.482	6.131	0.384	3.078	0.476	6.079	0.390
Hypertension	1.898	0.675	3.996	0.744	1.776	0.603	3.297	0.756	1.623	0.598	3.111	0.758
Hyperlipidemia	0.000	–	–	0.989	0.000	–	–	0.997	0.000	–	–	0.997
Drugs (No reference)												
NSAID	1.264	0.842	1.677	0.562	1.189	0.675	1.597	0.599	1.187	0.673	1.589	0.598
Systemic steroid	1.101	0.765	1.297	0.438	1.086	0.567	1.273	0.501	1.085	0.561	1.270	0.497

*HR* Hazard ratio, *CI* Confidence interval, *Adjusted HR* Adjusted variables listed in the table, *NSAID* Non-steroidal anti-inflammatory drugs

### Risk of collagen-associated adverse events

After adjusting for age, sex, catastrophic illness, low-income household, seasons, levels of urbanization and healthcare, the adjusted HR for collagen-associated adverse events in FQs group was 1.330 (95% CI; 0.778–2.276; *p* = 0.255). After multivariate analysis, we noticed that patient’s age was associated with the risk of GI perforation. As shown in Table 2, the risk of collagen-associate adverse effect was lower in patients aged 5~9 years old (aHR = 0.433; 95% CIs = 0.179–0.952; *p* = 0.040), patients aged 10~14 years old (aHR = 0.386; 95% CI = 0.163–0.909; *p* = 0.028).

### Subgroup analyses

The association of FQs with subsequent GI perforation was stratified by baseline demographic, catastrophic illness, low-income household, seasons, levels of urbanization and healthcare. Analyses of factors of GI perforation in 6-month tracking stratified by the aforementioned variables were performed by Cox regression as shown in Table 3. We did not observe any statistically significant difference between FQs group and FQs-free group after subgroup analyses of gender, age, catastrophic illness, low-income household, seasons, levels of urbanization and healthcare.

**Table 3:** Factors of gastrointestinal perforation in 6-month tracking stratified by variables listed in the table by using Cox regression

Fluoroquinolones	With			Without			Ratio	With vs. Without			
Stratified	Events	PDs	Rate (per 10^5^ PDs)	Events	PDs	Rate (per 10^5^ PDs)		Adjusted HR	95% CI	95% CI	*P*
Total	17	5,940,776.06	0.29	68	23,707,602.75	0.29	0.998	1.330	0.778	2.276	0.255
Gender											
Male	10	3,060,435.00	0.33	31	12,297,000.62	0.25	1.296	1.744	0.921	2.983	0.643
Female	7	2,880,341.06	0.24	37	11,410,602.12	0.32	0.749	1.008	0.556	1.724	0.297
Age group (years)											
< 1	1	183,600.00	0.54	4	820,422.12	0.49	1.117	1.503	0.876	2.572	0.675
1	2	256,265.94	0.78	3	876,927.19	0.34	2.281	2.279	0.998	4.027	0.052
2	1	286,506.19	0.35	4	1,024,530.50	0.39	0.894	1.194	0.702	1.960	0.536
3	2	325,930.75	0.61	5	1,282,879.44	0.39	1.574	1.822	0.943	2.894	0.071
4	1	384,465.19	0.26	6	1,542,322.12	0.39	0.669	0.899	0.525	1.275	0.489
5–9	3	1,677,499.81	0.18	14	6,622,008.94	0.21	0.846	1.139	0.669	1.949	0.376
10–14	2	1,521,852.94	0.13	10	6,123,547.69	0.16	0.805	1.080	0.635	1.851	0.447
≧15	5	1,304,655.25	0.38	22	5,414,964.75	0.41	0.943	1.271	0.742	2.170	0.285
Catastrophic illness											
Without	16	5,907,063.87	0.27	68	23,616,531.12	0.29	0.941	1.264	0.735	2.163	0.194
With	1	33,712.19	2.97	0	91,071.62	0.00	∞	∞	–	–	0.796
Low-income household											
Without	16	5,789,090.44	0.28	68	23,320,517.25	0.29	0.948	1.277	0.744	2.183	0.182
With	1	151,685.62	0.66	0	387,085.50	0.00	∞	∞	–	–	0.785
Season											
Spring	5	1,209,564.81	0.41	23	6,731,139.19	0.34	1.210	1.628	0.952	2.792	0.073
Summer	3	1,211,624.37	0.25	12	5,549,493.38	0.22	1.145	1.539	0.901	2.639	0.102
Autumn	2	1,596,290.69	0.13	10	5,377,612.87	0.19	0.674	0.907	0.524	1.551	0.773
Winter	7	1,923,296.19	0.36	23	6,049,357.31	0.38	0.957	1.288	0.751	2.204	0.288
Urbanization level											
1 (the highest)	6	1,305,977.19	0.46	30	6,700,633.87	0.45	1.026	1.378	0.808	2.364	0.134
2	2	2,119,226.31	0.09	12	8,911,079.56	0.13	0.701	0.942	0.553	1.616	0.446
3	7	1,142,905.31	0.61	19	3,835,392.81	0.50	1.236	1.663	0.971	2.843	0.059
4 (the lowest)	2	1,372,667.25	0.15	7	4,260,496.50	0.16	0.887	1.185	0.692	2.041	0.334
Level of care											
Hospital center	6	222,455.87	2.70	26	1,083,958.87	2.40	1.124	1.156	0.884	2.588	0.240
Regional hospital	5	343,936.38	1.45	19	1,737,590.94	1.09	1.329	1.794	0.972	3.059	0.062
Local hospital	4	281,046.94	1.42	15	1,249,182.12	1.20	1.185	1.589	0.932	2.735	0.179
Physician clinics	2	5,093,336.87	0.04	8	19,636,870.81	0.04	0.964	1.290	0.759	2.214	0.304

*PDs* Person-days, *Adjusted HR* Adjusted Hazard ratio: Adjusted for the variables listed in Table 2, *CI* Confidence interval

### Stratified by fluoroquinolones

Also, we did the subgroup analysis to confirm the association of FQs subgroup with subsequent GI perforation in different tracking periods by using Cox regression. In 3-day tracking period, we observed 2 events from FQs-free group. In 7-day tracking period, we observed 1 event from ofloxacin in the FQs group, and 6 events from FQs-free group. In 14-day tracking period, we observed 1 event from ciprofloxacin, 1 event from norfloxacin, and 1 event from ofloxacin in the FQs group, and 10 events from FQs-free group. In 28-day tracking period, we observed 1 event from ciprofloxacin, 1 event from norfloxacin, 2 events from ofloxacin in the FQs group, and 14 events from FQs-free group. In 3-month tracking period, we observed 1 event from ciprofloxacin, 3 events from norfloxacin, and 8 events from ofloxacin in the FQs group, and 45 events from FQs-free group. In 6-month tracking period, we observed 1 event from ciprofloxacin, 3 events from norfloxacin, and 13 events from ofloxacin in the FQs group, and 68 events from FQs-free group. The incidence rate and adjusted HR are listed in Table 4. None of them were statistically significant difference between FQs group and FQs-free group.

**Table 4:** Factors of gastrointestinal perforation stratified by fluoroquinolones subgroup in different tracking period by using Cox regression

Tracking period	Fluoroquinolones subgroup	Populations	Events	PDs	Rate (per 10^5^ PDs)	Adjusted HR	95% CI	95% CI	*P*	Adjusted HR	95% CI	95% CI	*P*
3-day	Without	133,684	2	23,707,602.75	0.01	Reference				Reference			
	With	33,421	0	5,940,776.06	0.00	0.000	–	–	0.995				
	Levofloxacin	653	0	112,735.69	0.00					0.000	–	–	0.960
	Ciprofloxaxin	1843	0	316,411.56	0.00					0.000	–	–	0.978
	Moxifloxacin	139	0	24,500.00	0.00					0.000	–	–	0.988
	Gemifloxacin	12	0	2160.00	0.00					0.000	–	–	0.998
	Norfloxacin	7041	0	1,261,596.87	0.00					0.000	–	–	0.996
	Ofloxacin	23,733	0	4,223,371.94	0.00					0.000	–	–	0.990
7-day	Without	133,684	6	23,707,602.75	0.03	Reference				Reference			
	With	33,421	1	5,940,776.06	0.02	0.512	0.080	27.426	0.756				
	Levofloxacin	653	0	112,735.69	0.00					0.000	–	–	0.982
	Ciprofloxaxin	1843	0	316,411.56	0.00					0.000	–	–	0.979
	Moxifloxacin	139	0	24,500.00	0.00					0.000	–	–	0.993
	Gemifloxacin	12	0	2160.00	0.00					0.000	–	–	0.986
	Norfloxacin	7041	0	1,261,596.87	0.00					0.000	–	–	0.978
	Ofloxacin	23,733	1	4,223,371.94	0.02					0.776	0.114	72.301	0.442
14-day	Without	133,684	10	23,707,602.75	0.04	Reference				Reference			
	With	33,421	3	5,940,776.06	0.05	1.025	0.044	1.356	0.111				
	Levofloxacin	653	0	112,735.69	0.00					0.000	–	–	0.986
	Ciprofloxaxin	1843	1	316,411.56	0.32					1.572	0.052	7.166	0.678
	Moxifloxacin	139	0	24,500.00	0.00					0.000	–	–	0.995
	Gemifloxacin	12	0	2160.00	0.00					0.000	–	–	0.986
	Norfloxacin	7041	1	1,261,596.87	0.08					1.342	0.020	6.988	0.501
	Ofloxacin	23,733	1	4,223,371.94	0.02					0.427	0.035	4.972	0.494
28-day	Without	133,684	14	23,707,602.75	0.06	Reference				Reference			
	With	33,421	4	5,940,776.06	0.07	1.137	0.090	1.498	0.157				
	Levofloxacin	653	0	112,735.69	0.00					0.000	–	–	0.978
	Ciprofloxaxin	1843	1	316,411.56	0.32					1.266	0.028	2.999	0.267
	Moxifloxacin	139	0	24,500.00	0.00					0.000	–	–	0.978
	Gemifloxacin	12	0	2160.00	0.00					0.000	–	–	0.964
	Norfloxacin	7041	1	1,261,596.87	0.08					2.052	0.184	22.485	0.532
	Ofloxacin	23,733	2	4,223,371.94	0.05					0.307	0.555	1.752	0.188
3-month	Without	133,684	45	23,707,602.75	0.19	Reference				Reference			
	With	33,421	12	5,940,776.06	0.20	1.049	0.156	1.925	0.198				
	Levofloxacin	653	0	112,735.69	0.00					0.000	–	–	0.952
	Ciprofloxaxin	1843	1	316,411.56	0.32					1.076	0.013	1.972	0.129
	Moxifloxacin	139	0	24,500.00	0.00					0.000	–	–	0.978
	Gemifloxacin	12	0	2160.00	0.00					0.000	–	–	0.986
	Norfloxacin	7041	3	1,261,596.87	0.24					1.472	0.402	5.411	0.524
	Ofloxacin	23,733	8	4,223,371.94	0.19					0.390	0.145	1.786	0.379
6-month	Without	133,684	68	23,707,602.75	0.29	Reference				Reference			
	With	33,421	17	5,940,776.06	0.29	1.330	0.778	2.276	0.255				
	Levofloxacin	653	0	112,735.69	0.00					0.000	–	–	0.975
	Ciprofloxaxin	1843	1	316,411.56	0.32					1.402	0.197	10.121	0.702
	Moxifloxacin	139	0	24,500.00	0.00					0.000	–	–	0.966
	Gemifloxacin	12	0	2160.00	0.00					0.000	–	–	0.973
	Norfloxacin	7041	3	1,261,596.87	0.24					0.911	0.348	3.526	0.813
	Ofloxacin	23,733	13	4,223,371.94	0.31					1.442	0.793	2.601	0.199

*PDs* Person-days, *Adjusted HR* Adjusted hazard ratio: Adjusted for the variables listed in Table 2, *CI* Confidence interval

## Discussion

Fluoroquinolones are highly effective antimicrobial agents with the following advantages: a broad spectrum of bactericidal activity, ideal bioavailability, both oral and intravenous formulations, high serum levels and a large volume of distribution. These advantages increase the FQs usage in a wide variety of infectious diseases, including skin and respiratory infections for adults. However; the post-marketing surveillance data indicates that FQs may cause subsequent collagen-associated adverse events. Since year 2008, the U.S. FDA has declared several warnings about the association of FQs with disabling and potentially permanent side effects involving tendons, muscles, joints, nerves and the central nervous system in succession [28]. Additionally, due to the toxic effects observed from juvenile animals treated with FQs, the use of FQ-related drugs became rather limited in pediatric population [13]. Only ciprofloxacin and levofloxacin are approved by the U.S. FDA for the treatment of inhalation anthrax, complicated UTIs, and pyelonephritis in children aged 1 to 17 years old [15]. Furthermore, with the overuse of antimicrobial agents in clinical practice, the emergence of antibiotic resistant bacteria is rising in Taiwan [29]. To overcome the surge of drug resistant issues, the use of FQs is also increasing. To evaluate the safety issue of FQs, it is necessary for us to investigate serious collagen-associated adverse events in pediatric population.

The main findings of our study demonstrated that there was no difference in the risk of collagen-associated adverse events between FQs group and FQs-free group in pediatric population. By reviewing published studies [3–7, 18], we noticed that the reported higher risk of collagen-associate adverse effect in FQs-related cases were found in patients at the age of over 18 years old. However, most of these studies were designed in case-control studies and the subjects were patients older than 18 years old [3–7, 18]. After comparing the difference between our findings and previous reported results, we made the following to several explanations:

(i) The study design: the subjects from case-control studies were identified by evaluating the outcome status at the outset of the investigation [4, 5, 18]. Enrolled cases with the outcome of interest are matched with a control group without it. The major and inevitable problems in case-control studies are the sampling bias, observation bias and recall bias. In contrast, cohort studies are usually used to confirm the disease incidence, causes, and prognosis. Cohort studies are often utilized for measuring events in chronological order and clarifying the relationship between cause and effect [30, 31]. Consequently, we designed our study in the form of cohort study to assess the association between use of FQs and effect of collagen-associated adverse events. We believe that this cohort study provides more objective and reliable information than case-control studies.
(ii) Dosage adjustment and rigorous monitoring in pediatric population: dosage of FQs is calculated and adjusted for by body weight in children before prescriptions. Maximum dosage limitation is advised by clinical guideline and FDA [15]. Meanwhile, healthcare providers usually follow the recommendation strictly because of the concern of the adverse effects to this vulnerable population. Furthermore, physicians tend to avoid this class of antimicrobial agents in clinical practice and preserve them as the final therapy for difficult and serious bacterial infections. Therefore, the side effects become much less as expected attributing to precise dosage and strict indication.
(iii) Comorbidity in elderly population: with regard to most of the FQs studies [3–7, 18], the enrolled subjects were older than 18 years old, especially the elderly population with comorbidities, like cardiovascular diseases and diabetes [32]. For example, cardiovascular diseases such as hypertension and hyperlipidemia, make the blood vessels more vulnerable and subsequently increase the risk of aortic aneurysms [33]. Patients with diabetes have more problematic blood vessels and retinopathy, which increases the risk of retinal detachments [34, 35]. Thus, these underlying diseases may contribute to the side effects of FQs to some extent in elderly population, but they are less frequent in younger generation. These observative findings may partially explain why we found that pediatric patients in our cohort have less side effects compared with previous reports. Since these comorbidities were adjusted as the covariates in our study, we believe that these items won’t be the confounding factors in this cohort.

Though our findings differ from the previous studies, we believe that it reflects the real clinical situations in pediatric population by meticulously analyzing the database resource in Taiwan. The National Health Insurance Research Database (NHIRD) of Taiwan, which covers more than 99.6% of the Taiwanese population, makes our study results more statistically powerful and representative [21]. NHIRD has been also widely used in generating evidences to support clinical decisions or healthcare policy-making [22].

Several limitations of the study should be noted. First, we are not sure about the compliance of the outpatient department patients because some of them may discontinue oral form of FQs abruptly [36]. It may cause underestimation of the adverse effects of FQs because the patients did not complete the treatment course. According to the results from previous studies, up to 52.7% of subjects reported that they did not precisely follow the physicians’ advice about antibiotics use [37]. Second, the clinical conditions and underlying diseases of the enrolled cases during the observation period were not available. Underlying illness and chronic diseases may predispose to develop the collagen-associated adverse effects significantly. Third, no supportive image reports and laboratory data from each medical record to confirm the diagnosis of collagen-associated adverse effects, we defined the outcome in this study by reviewing the registration of ICD-9-CM codes. The potential misclassification bias cannot be ruled out in our study [38]. Despite the potential limits, our study also has some strengths because the results of this study are based on a real-world database analysis. The enormous number of prescriptions records makes the results more reliable and unbiased. In addition, we tracked the patient’s outcome up to 6 months to see the long-term impact of the FQs exposure.

## Conclusions

We designed this population-based, retrospective cohort study to evaluate the safety of FQs in pediatric population in Taiwan. In this study, we did not observe any statistically significant difference in the incidence of collagen-associated adverse events between FQs group and FQs-free group. FQs use in pediatric patients seems safe under instruction on the basis of our findings. Therefore, the risk of collagen-associated adverse events in pediatric population may be overestimated in previous studies. Since physicians still need FQs to treat serious and fulminant bacterial infections in children, our data supports that physicians may prescribe FQs in seriously infected young patients as indicated in the guideline.

## Data Availability

The data that support the findings of this study are available from Health and Welfare Data Science Center, Ministry of Health and Welfare (HWDC, MOHW) of Taiwan but restrictions apply to the availability of these data, which were used under license for the current study, and so are not publicly available.

## Abbreviations

aHR: Adjusted hazard ratio
CI: Confidence interval
FQ: Fluoroquinolones
HR: Hazard ratio
ICD-9-CM: International Classification of Diseases, Ninth Revision, Clinical Modification
NHI: National Health Insurance
NHIRD: National Health Insurance Research Database
PDs: Person-days
